# Effect of the Relative Timing between Same-Polarity Pulses on Thresholds and Loudness in Cochlear Implant Users

**DOI:** 10.1007/s10162-020-00767-y

**Published:** 2020-08-24

**Authors:** François Guérit, Jeremy Marozeau, Bastian Epp, Robert P. Carlyon

**Affiliations:** 1 Hearing Systems Group, Department of Health Technology, Technical University of Denmark, 352 Ørsteds Plads, 2800 Kgs. Lyngby, Denmark; 2 Present Address: Cambridge Hearing Group, MRC Cognition and Brain Sciences Unit, University of Cambridge, 15 Chaucer Road, Cambridge, CB2 7EF United Kingdom

**Keywords:** Polarity, Cochlear implants, Inter-phase gap, Inter-pulse interval, Facilitation

## Abstract

The effect of the relative timing between pairs of same-polarity monophasic pulses has been studied extensively in single-neuron animal studies and has revealed fundamental properties of the neurons. For human cochlear implant listeners, the requirement to use charge-balanced stimulation and the typical use of symmetric, biphasic pulses limits such measures, because currents of opposite polarities interact at the level of the neural membrane. Here, we propose a paradigm to study same-polarity summation of currents while keeping the stimulation charge-balanced within a short time window. We used pairs of mirrored pseudo-monophasic pulses (a long-low phase followed by a short-high phase for the first pulse and a short-high phase followed by a long-low phase for the second pulse). We assumed that most of the excitation would stem from the two adjacent short-high phases, which had the same polarity. The inter-pulse interval between the short-high phases was varied from 0 to 345 μs. The inter-pulse interval had a significant effect on the perceived loudness, and this effect was consistent with both passive (membrane-related) and active (ion-channel-related) neuronal mechanisms contributing to facilitation. Furthermore, the effect of interval interacted with the polarity of the pulse pairs. At threshold, there was an effect of polarity, but, surprisingly, no effect of interval nor an interaction between the two factors. We discuss possible peripheral origins of these results.

## Introduction

Cochlear implants (CIs) treat cases of severe-to-profound sensorineural hearing loss by electrically stimulating the spiral ganglion neurons (SGNs). There is a large variability of outcomes across users, with a significant amount of this variability accounted for by the duration of deafness prior to implantation (Blamey et al. 2013). This likely reflects the importance of physiological changes along the auditory pathway following years of deafness.

Amongst the first stages of the auditory pathway, both the myelination and the diameter of the spiral ganglion neurons (SGNs) can decrease following sensorineural hearing loss (Leake and Hradek 1988; Nadol 1997). These morphological changes affect how the SGNs integrate the electrical charge delivered by CIs (Bostock et al. 1983; Colombo and Parkins 1987; Smit et al. 2008; Resnick et al. 2018). This is because the passive behaviour of the neuronal membrane is that of a leaky integrator (Lapicque 1907), and both the diameter and the amount of myelination can strongly affect its capacitive-resistive properties.

Spike generation at the level of the SGNs is driven not only by passive, sub-threshold leaky integration but also by sub- and supra-threshold active mechanisms (i.e. the ion channels). It is possible that years of sensorineural hearing loss will affect such ion channel dynamics. Furthermore, any changes in these mechanisms will likely have consequences on the integration of multiple electrical pulses via facilitation, refractoriness, accommodation and long-term adaptation (cf. Boulet et al. 2016).

Single-neuron animal studies typically characterize properties of charge integration with pairs of monophasic pulses, and vary the inter-pulse interval (IPI), while comparing the response to that of a single pulse (Lucas 1910; Dynes 1996; Cartee et al. 2000, 2006). The use of monophasic (purely anodic or cathodic) pulses is precluded in humans, because unbalanced electrical stimulation can damage the electrode contacts and create ototoxic products (Lilly et al. 1955; Brummer and Turner 1977; Merrill et al. 2005). Charge balancing is usually achieved by stimulating with short (~ 50 to 200 μs) symmetric biphasic pulses, consisting of an anodic and a cathodic phase of equal amplitude and duration. Several human studies have investigated the effect of IPI with pairs of such charge-balanced biphasic pulses. This includes physiological measures such as functions measuring the recovery of the neural response to the second pulse as a function of the IPI (e.g. Abbas and Brown 1991; Brown et al. 1996; Morsnowski et al. 2006). In addition, psychophysical studies have measured thresholds and most comfortable levels as a function of the IPI and of the relative level between two pulses (Pfingst et al. 1996; McKay and McDermott 1998; Nelson and Donaldson 2001; de Balthasar et al. 2003; McKay et al. 2013; Karg et al. 2013; Macherey et al. 2017; Guérit et al. 2018). However, when it comes to linking the results of these studies to the neural mechanisms of spike generation and particularly the effects of auditory deprivation, the clinical use of symmetric biphasic pulses complicates interpretation. This is because the two nearest phases are necessarily of opposite polarity and so partially cancel each other when integrated by the cell membrane (e.g. van den Honert and Mortimer 1979). Furthermore, both anodic and cathodic phases of biphasic pulses can be excitatory, probably by eliciting spikes at different portions of the SGN, as suggested in animals (Miller et al. 1999a) and humans (Macherey et al. 2008; Undurraga et al. 2013). Complex order effects can stem from this ability of both polarities to generate spikes, depending on the relative ratio of current and neural excitation from each phase (Guérit et al. 2018). The situation is further complicated by the so-called rebound spikes that can be generated by the offset of the phase of a biphasic pulse that hyperpolarizes the nerve membrane (also called “anode break excitation”, Hodgkin and Huxley 1952). Having a paired pulse paradigm that limits opposite-polarity interactions could therefore improve the characterization of the temporal mechanisms of spike generation in human CI users.

**Fig. 1:**
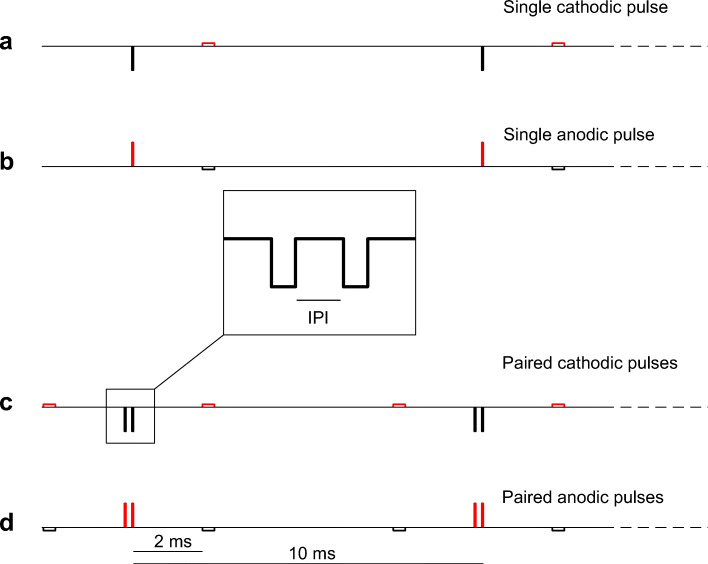
Schematic representation of the different conditions. **A**–**B** “Single” pseudo-monophasic pulses (asymmetry ratio of 8), with the short-high phase being cathodic (**A**) or cathodic (**B**). The inter-phase gap was 2-ms long, and the pulses were repeated at a rate of 100 Hz for 400 ms. **C**–**D** “Paired pulses”. For the paired pulses, the inter-pulse interval (IPI) had values ranging from 0 to 344 us

Here we propose and test a paired pulse paradigm approximating that used with monophasic pulses in animals (Cartee et al. 2000, 2006). The proposed paradigm uses asymmetric, pseudo-monophasic and charge-balanced pulses (Fig. 1), each consisting of one long-low and one short-high phase. The underlying assumption is that the short-high phase will be more efficient in eliciting a response than the long-low one because the neural membrane behaves approximately as a leaky integrator at sub-threshold levels (ignoring here active mechanisms as a first approximation; Lapicque 1907; Miller et al. 2001; Undurraga et al. 2013). Furthermore, inserting a gap of 2 ms between the short-high and long-low phases should avoid between-phase integration effects at the level of the membrane. As a further test for any influence of the long-low phases on loudness, we repeated our loudness balancing experiments with a subset of listeners with the asymmetry ratio of the pseudo-monophasic pulses increased from 8 to 16. The reasoning was that any effect of the long-low phases would be reduced by the halving of their amplitude, despite the doubling of their duration.

As in the studies on animal models by Cartee et al. (2000) and by Bierer and Middlebrooks (2004), we expected that short IPIs between two short-high phases of the same polarity would lead to “summation”, i.e. sub-threshold integration of charge at the level of the membrane. We therefore investigated whether short IPIs reduced detection thresholds and increased loudness. We probed the time course of this effect by varying the IPI in conditions where the two short-high phases were anodic or cathodic. We included a 0-μs IPI condition, allowing us to study the effects of doubling the phase duration without the influence of a temporally adjacent equal-amplitude phase of opposite polarity.

One of the reasons for including conditions with both polarities is that it has been suggested that anodic and cathodic polarities might target preferentially the central and peripheral axons, respectively (Miller et al. 1999a; Rattay et al. 2001). These two axons of the SGNs might differ in their amount of myelination, diameter and degeneration following sensorineural hearing loss. The distance between the nodes of Ranvier, as well as the distance between the nodes and the highly capacitive soma, might also differ between the peripheral and central axons (Liberman and Oliver 1984). For example, modelling studies suggest that peripheral axons should exhibit longer time constants of passive, sub-threshold charge integration compared with the central axons (Cartee 2000, 2006; Joshi et al. 2017). We, therefore, expected our results to show signs of integration at longer IPIs with cathodic currents, when compared with anodic currents.

## Methods

### Listeners

Six listeners took part, all of whom were recipients of an advanced bionics CI (cf. Table 1 for demographics). Listeners were recruited both in Cambridge (UK) and Copenhagen (Denmark), and the experimental procedure was approved, respectively, by the National Research Ethics Committee for the East of England (ref. number 00/327) and the Danish Science-Ethics Committee (ref. number H-16036391). All listeners signed a participation agreement before data collection.

**Table 1:** Demographics of the CI listeners. All listeners were implanted with an Advanced Bionics HiRes90k device. 1j is a straight array, ms (“mid-scala”) is curved. Subject codes given in parentheses are those used for the same subjects in other publications from our two laboratories (S1 and AB2, Guérit et al. 2018, AB2 and AB13 in Archer-Boyd et al. 2018)

Subject ID	Age (y)	Duration of implant use (y)	CI Side	Electrode used for testing	Type of electrode array	Aetiology
S1 (S1)	57	3	Right	9	ms	Meniere’s
S2	60	9	Left	9	1j	Pendred syndrome
S3	28	4	Left	8	ms	Unknown, pre-lingual
S4 (AB13)	76	9	Right	9	1j	Unknown
S5 (AB2)	57	9	Left	9	1j	Ototoxicity
S6	66	3	Right	9	ms	Rhesus disease

### Setup and Stimuli

We conducted all experiments by means of direct stimulation, i.e. using research hardware (CPI-II clinical interface, PSP speech processor) and software (BEDCS 1.18, PPS toolbox, Matlab 2014a) instead of the clinical speech processor of the listeners.

Stimuli consisted of 400-ms trains of 100-pps pseudo-monophasic pulses. Each pseudo-monophasic pulse (Fig. 1A and 1B) consisted of a short-high and a long-low phase, separated by a gap of 2 ms. The duration of the short-high phase was 43 μs and that of the long-low phase was eight times longer, and with the amplitude reduced by the same factor. In the “single cathodic” and “single anodic” conditions, the short-high phase was cathodic (Fig. 1A) and anodic (Fig. 1B), respectively. For a subset of listeners, the loudness-balancing measures were repeated with the long-low pulse 16 times longer and 1/16th the amplitude of the short-high phase.

We also created trains of paired pulses where the long-low phase preceded the short-high phase for the first pulse, but followed it for the second pulse (Fig. 1C and D). That way, the two short-high phases (which we assumed would create most of the neural response) were temporally adjacent. Paired pulse stimuli had inter-pulse intervals (IPI) ranging from 0 to 345 μs. At 0-μs IPI, the design was such that there was no glitch in the amplitude between the two pulses. In a similar manner as for the single pulse stimuli, we created a cathodic (Fig. 1C) and anodic version (Fig. 1D), with the short-high phases being cathodic and anodic, respectively.

Prior to and throughout the experiments, we checked the stimuli with a test implant (HiRes90k) and a digital storage oscilloscope. Asymmetric, pseudo-monophasic pulses are charge-balanced, but only within the limits of compliance of the device (7–8 V, Mesnildrey 2017): above those, the short-high phase would not reach its assigned amplitude, and charge balancing would rely on the blocking capacitors of the device. We therefore measured impedances at the beginning and the end of each session. Across all listeners and sessions, the maximum voltage we reached was 4.9 V, and we did not see any significant changes compared to the start of the session.

### Detection Thresholds

Detection thresholds for all conditions were measured with a one-up-three-down two-alternative forced-choice procedure (Levitt 1971). We tracked the levels on a logarithmic (dB) scale and used eight reversals, two with a step size of 1 dB, followed by six with a step size of 0.25 dB. The actual levels could differ slightly from the desired levels. This is because the HiRes90k device dynamic range is divided in a linear, not logarithmic way and because the minimum achievable step size depends on the dynamic range used (1 μA between 0 and 255 μA, 2 μA for 0–510, 4 μA for 0–1020, 8 μA for 0–2040). Asymmetric pulses further limit the minimum step size achievable, because of the need to code accurately the level of the long-low phase (and hence keep the asymmetry ratio constant across the dynamic range). With a ratio of 8, measurements with a test implant and an oscilloscope revealed that the minimum step size had to be doubled (2 μA for 0–255 μA, etc.) in order to ensure a constant asymmetry ratio. We therefore computed the final thresholds from the actual levels of the last six reversals, not the desired or tracked levels. Each measurement was repeated twice, leading to 24 measurements (for each polarity: single pulse, paired pulses with 0-, 43-, 86-, 172- and 345-us IPIs). We ensured that the starting point of every trial was clearly audible.

### Loudness Balancing at Most Comfortable Levels (MCLs)

For all conditions (single and paired pulses at all gaps, for both polarities), we obtained the most comfortable levels (MCLs) using an 11-point loudness scaling chart (number 6 corresponded to the MCL). We then picked a level slightly below the MCL of the single cathodic pulse as a reference for the subsequent loudness balancing. We did not pick the MCL itself as a reference in order to give enough headroom for the loudness balancing procedure without reaching any uncomfortable loudness.

For each loudness balancing, the subject heard two sounds and reported which of the two was the louder. The level of the first sound was fixed, and the experimenter adjusted the level of the second sound until both had the same loudness, bracketing several times around this point of subjective equality. The adjustable sound was then fixed at this level and the roles of the fixed and adjustable sounds were switched, and a second adjustment was performed. This was repeated an additional two times, again switching the first and second sound for each adjustment. The final balanced value was computed from the average of the four adjustments (in dB re 1 μA).

We first matched the level of the single anodic pulse to the reference (single cathodic pulse). The listeners then balanced the loudness of the paired pulse with 0-μs IPI to that of the single pulse, for each polarity at a time. Next, the paired pulse with 43-μs IPI was balanced to the paired pulse with 0-μs IPI, 86 to 43-μs IPI and finally 172 to 86-μs IPI. The paired pulses with 345-μs IPI were not included to ensure that the loudness balancing for all anodic or cathodic conditions would fit within one testing session.

## Results

### Single Pulses

**Fig. 2:**
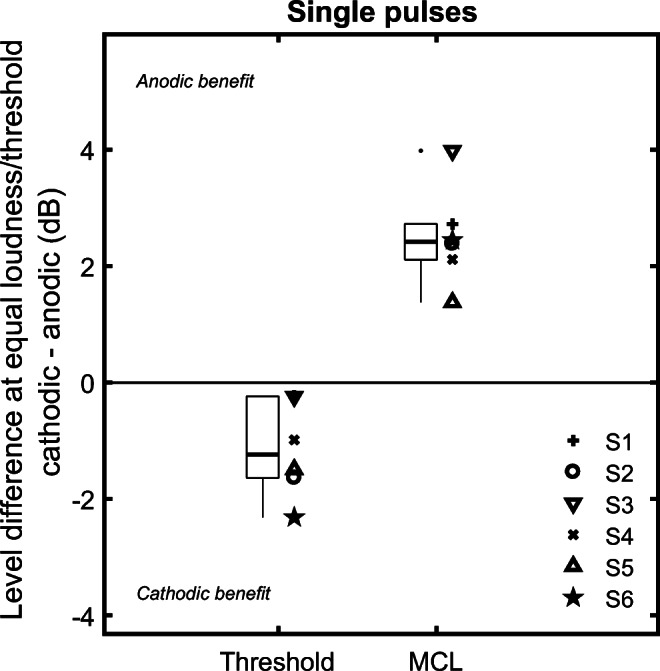
Polarity difference in detection thresholds and loudness-balanced MCLs for the single pulses. Lower and upper limits of the boxes: 25th and 75th percentiles. Horizontal black line: median level. Whiskers: 25th (or 75th) percentile minus (or plus) 1.5 the interquartile range. Dots correspond to data points with values outside the range delimited by the whiskers

Detection thresholds and MCLs for the anodic single pulses were on average 43.1 and 51.2 dB re 1 μA, respectively. Figure 2 shows the difference in threshold and MCL between anodic and cathodic pulses for each listener. At MCL, the anodic stimuli required less current than cathodic stimuli to achieve the same loudness (+ 2.50 dB, paired *t* test, t(5) = 7.16, *p* < 0.001), leading to a positive polarity effect, defined as the cathodic MCL minus the anodic MCL. In contrast, at threshold less current was required for cathodic stimuli than for anodic stimuli (− 1.15 dB, t(5) = 3.41, *p* = 0.019), leading to a small negative polarity effect. In both cases, the effect was in the same direction for all listeners.

### Paired Pulses

**Fig. 3:**
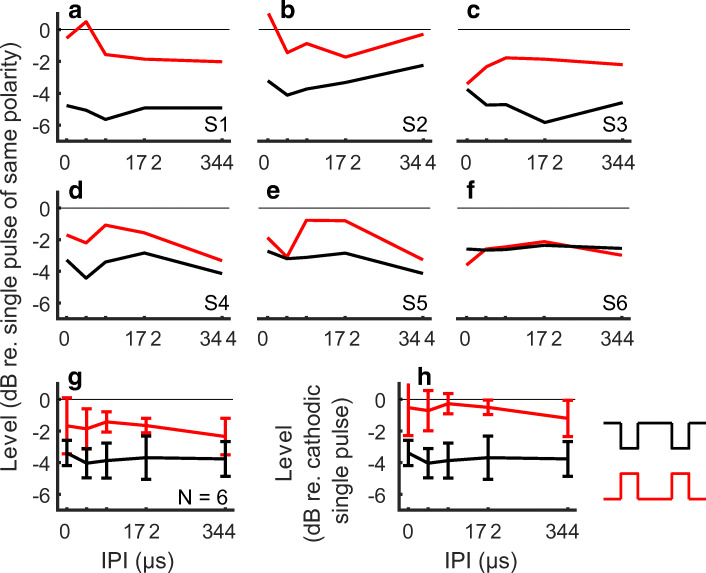
Detection thresholds for the anodic (red) and cathodic (black) paired pulses. **A**–**F** Individual results, with the values normalized to the threshold of the single pulse with corresponding polarity. **G** Mean and standard deviation of the results shown in panels A to F. **H** Mean and standard deviation across listeners, with the values normalized to the threshold of the single cathodic pulse

Figure 3 shows the individual and the mean group detection thresholds with paired pulses. For panels A to G, levels are normalized to the level of the threshold for the single pulse of the same polarity. A repeated measures ANOVA showed an effect of polarity (F(1, 5) = 11.5, *p* = 0.0195), but no effect of IPI (F(4, 20) = 0.77, *p* = 0.56) nor an interaction between polarity and IPI (F(4, 20) = 1.1, *p* = 0.40). When pooled across IPIs, the anodic pulses (red line and symbols) required on average 1.80 dB less current than single pulses to reach threshold. For cathodic pulses (black line and symbols), this reduction was significantly larger (t(5) = 3.39, *p* = 0.0194) and amounted to 3.76 dB. Because thresholds were lower for single cathodic pulses than for single anodic pulses, the absolute levels of the pulse pairs at threshold are also lower for the cathodic stimuli at all IPIs (panel H in Fig. 3).

**Fig. 4:**
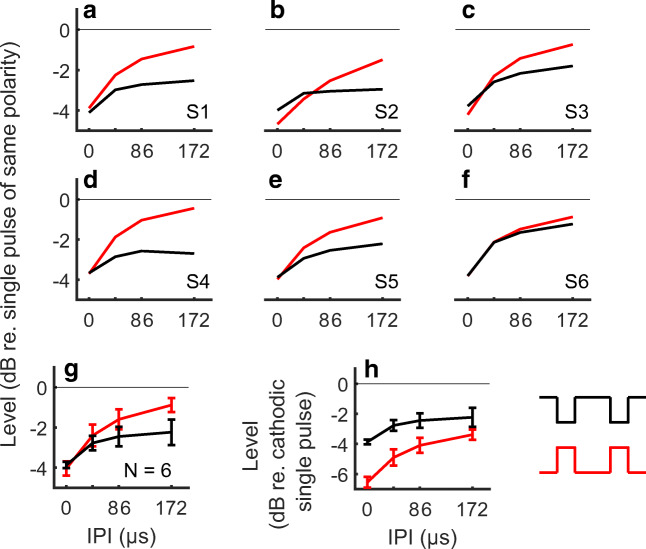
Loudness-balanced levels for the anodic (red) and cathodic (black) paired pulses. **A**–**F** Individual results, with the values normalized to the level of the single pulse with corresponding polarity. **G** Mean and standard deviation of the results shown in panels A to F. **H** Mean and standard deviation across listeners, with the values normalized to the level of the single cathodic pulse at MCL

Parts A–G of Fig. 4 show the individual and mean group results of the loudness balancing for the paired pulses, relative to the single-pulse MCL for the same polarity. Unlike the case for thresholds, all subjects show a consistent and monotonic increase in MCL with increasing IPI. A repeated-measures ANOVA on the (normalized) levels shown in panels A to G of Fig. 4 showed significant effects of polarity (F(1, 5) = 12.7, *p* = 0.0162), IPI (F(3, 15) = 326, *p* < 0.001) and a significant interaction between polarity and IPI (F(3, 15) = 26.7, p < 0.001). The interaction reflects the more gradual and smaller effect of IPI on the MCLs for cathodic than for anodic pulses.

Post hoc *t* tests with Bonferroni corrections applied to the data in panels A to F of Fig. 4 showed that the difference between the two polarities was significant only at IPIs of 86 and 172 μs (0 μs: t(5) = 1.3, *p* = 0.25; 43 μs: t(5) = 1.97, *p* = 0.106; 86 μs: t(5) = 4.22, *p* = 0.0083; 172 μs: t(5) = 5.20, *p* = 0.0035). At 0 μs (equivalent to doubling the phase duration of the single pulse), an average level reduction of 3.96 dB was needed to achieve the same loudness as the single pulses (both polarities pooled together, 4.04 and 3.86, respectively, for anodic and cathodic pulses). Part H of Fig. 4 shows the average MCLs for the anodic and cathodic pulse pairs relative to the MCL for the single cathodic pulse. It can be seen that the MCL for the anodic pulse pair is now 2.5 dB lower than for the cathodic pulse pair at an IPI of 0 μs (because of the 2.5 dB difference between the loudness of the single pulses) and that this difference decreases to about 0.8 dB at an IPI of 172 μs.

**Fig. 5:**
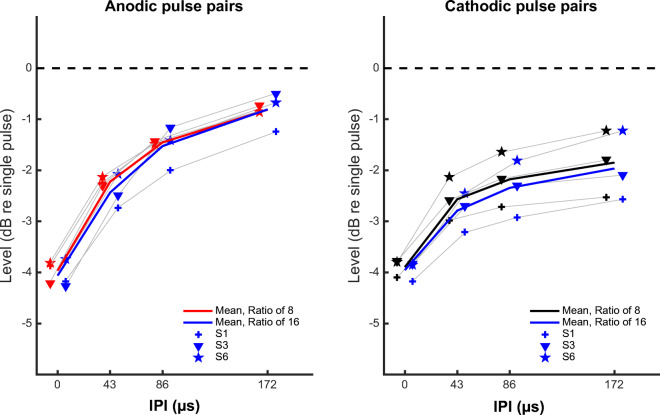
Loudness-balanced levels for the anodic (left panel) and cathodic (right panel) paired pulses, re-plotted from Fig. 4. Blue lines and symbols show the values obtained when re-testing with an asymmetry ratio of 16. Thick lines indicate the mean value across subjects. For visibility, the individual anodic and cathodic results are slightly shifted from their actual IPI value

The results for three listeners tested with the asymmetry ratio increased to 16 are shown in Fig. 5. Those results are very similar to those obtained with a ratio of 8, thereby providing no indication of an effect of the long-low phases on loudness.

## Discussion

Paired pulses required less current than single pulses to elicit the same loudness. This effect was more pronounced at the shortest IPIs and depended on the polarity of the stimulus. The level of the paired anodic pulses was only 0.9 dB lower than that of the single anodic pulse at the largest IPI tested (172 μs). In contrast, at this IPI a larger difference of 2.2 dB was observed between the MCL of the paired cathodic pules relative to the single cathodic pulse. No clear effect of IPI occurred at threshold. In the following, we discuss mechanisms that could explain those results, including interactions at the level of the neural membrane and in more central processes involved in loudness integration.

### Single Pulses

For single pulses, changing the polarity had a significant effect on both MCLs and thresholds.

At MCLs, the single anodic pulse required on average 2.50 dB less current than the single cathodic pulse to achieve the same loudness. This is consistent with most previous studies using pseudo-monophasic, triphasic and quadraphasic pulses (Macherey et al. 2008, 2017; van Wieringen et al. 2008; Bahmer et al. 2010, 2017; Undurraga et al. 2013; Carlyon et al. 2013; Guérit et al. 2018; Jahn and Arenberg 2019a, b), all of which used short inter-phase gaps of less than 10 μs. Hence, the finding of lower MCLs for anodic than for cathodic pulses appears to generalize to stimuli with the longer 2-ms inter-phase gap used here. Indeed, Macherey et al. (2008) found a significant polarity effect of 1.8 dB between pseudo-monophasic anodic and cathodic pulses, with an inter-phase gap as long as 6.4 ms. However, in a study that used a 4.7-ms inter-phase gap between the short-high and long-low phases of pseudo-monophasic pulses (Macherey et al. 2006), there was no difference in MCL between anodic and cathodic pulses. That study used a longer phase duration of 97 μs than used here (43 μs) or by Macherey et al. (2008) (22 μs). Hence, it may be that the existence of a polarity effect with long IPGs requires a short-phase duration. Note that modelling from Miller et al. (2001) suggests that short-phase durations (40 μs and below) are better suited for the use of pseudo-monophasic pulses, because it increases the difference in efficiency between the short-high and long-low phases.

In contrast to the MCL results, cathodic pulses required on average 1.15 dB less current than anodic pulses to reach threshold. The direction of this effect was the same for all listeners. Other studies have usually reported no consistent effect of polarity on detection thresholds (Macherey et al. 2006; Undurraga et al. 2013). Polarity effects at threshold can however occur on an individual, electrode-to-electrode basis (Carlyon et al. 2018; Goehring et al. 2019; Jahn and Arenberg 2019a, b; Mesnildrey et al. 2020). It might be that with another combination of electrodes and listeners, we would see different effects in our results at threshold. It might also be that our results are due to the rather long gap between the short-high and long-low phases (2 ms). Indeed, as discussed in the following sections, this might allow for both phases to contribute at threshold.

### Mechanisms Underlying the Effect of Polarity and IPI on Thresholds

A common assumption with pseudo-monophasic pulses is that the short-high phase creates most of the neural response (Miller et al. 2001). Recordings of electrically evoked auditory brainstem responses (eABR) support this assumption in humans, as they show a large response only to the short-high phase (Undurraga et al. 2013, with similar parameters to this study). Our results with paired pulses at MCL also support this assumption, as the loudness interacted strongly with the IPI between the two short-high phases. Furthermore, we found very similar results, in a subset of listeners, when the asymmetry ratio was increased to 16. In contrast, detection thresholds for the paired pulses showed no effect of IPI nor an interaction between IPI and polarity. Detection thresholds only exhibited an overall decrease for paired pulses, when compared with single pulses. This decrease was significantly larger for cathodic (− 3.8 dB) than anodic paired pulses (− 1.8 dB).

**Fig. 6:**
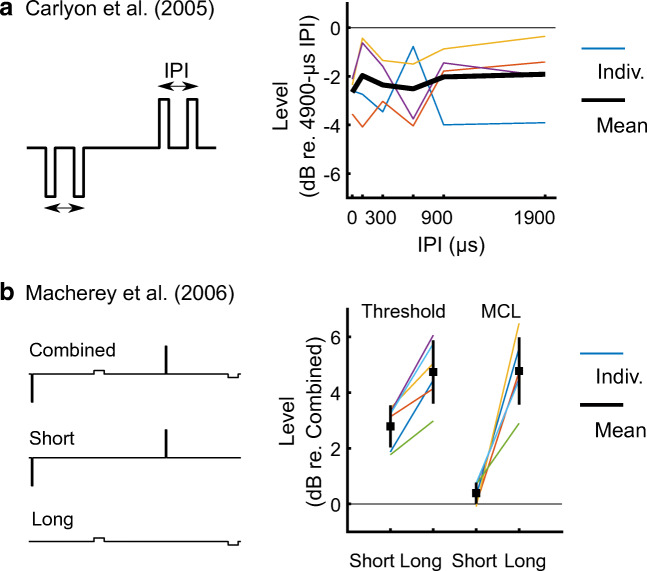
**A** Redrawn data and stimulus from Carlyon et al. (2005). The pictogram of the pulses is not to scale to be able to see the IPI. The rate of stimulation was 100 Hz, equivalent to 10 ms between the first pulses of each pulse pair. Pulses were presented in bipolar mode, phase duration was 100 μs, IPIs ranged from 0 to 1900 μs, only detection thresholds were measured. **B** Redrawn data and stimuli from Macherey et al. (2006), with a true scaling of the pulses. The short- and long-phase durations were 21.6 μs and 172.4 μs, respectively. The presentation rate was 407 Hz, equivalent to approximately 2.5 ms between each short-high phase (as well as between each long-low phase). Levels are re-drawn relative to the level of the combined stimulus at threshold or MCL (Fig. 10 in original manuscript). Thick black error bars indicate the mean ± 1 standard deviation across 6 participants

Our results at detection threshold are consistent with those of Carlyon et al. (2005), whose stimuli and results are redrawn in Fig. 6, panel A. They presented CI users with pairs of same-polarity monophasic pulses in bipolar mode. These alternated in polarity at every pair presentation: each pair of anodic pulses was followed by a pair of cathodic pulses, at a rate of 100 Hz. The alternation of polarity and the use of a bipolar mode do not allow for any polarity-specific interpretation of their results. However, similar to our results at threshold, they showed no effect of varying the IPI on detection thresholds, for intervals ranging from 0 to 1900 μs. That study did not report any MCLs, and it is unknown whether there was a strong effect of IPI at MCL, as shown in our results. Pulse pair facilitation at detection threshold and short IPIs has been observed in studies by Karg et al. (2013) and de Balthasar et al. (2003). Both studies however used paradigms with both pulses being symmetric biphasic pulses. There might therefore have been complex opposite-polarity interactions happening in their study: for example, it is unclear which of the four phases of these paradigms (anodic-cathodic then cathodic-anodic or vice-versa) contributes, and if their relative contribution changes as a function of IPI.

The absence of an effect of IPI at threshold might not only stem from the absence of an interaction between the short-high phases but might also indicate a significant contribution from the long-low phases at threshold. A contribution of the long-low phases at threshold is consistent with the results of Macherey et al. (2006), redrawn in Fig. 6, panel B. In one condition, they decomposed their alternating-polarity, pseudo-monophasic pulses into stimuli consisting of either the long-low phases or the short-high phases alone. The combined stimulus had lower detection thresholds than both the trains of short-high and long-low phases, consistent with both the long-low and short-high phases contributing to threshold. This contrasted with the results obtained at MCL, where the loudness of the combined stimulus was similar to that of the short-high phases only. Hence, it is possible that, for our stimuli also, the long-low phases influenced threshold but not MCL, thereby leading to an effect of inter-pulse interval only at MCL. However, this cannot completely explain the lack of effect of inter-pulse interval on thresholds because, as noted above, Carlyon et al. (2005) observed a similar result in a paradigm that did not include these long-low phases.

Overall, our results are consistent with the previous studies both at MCL and at detection threshold. The fact that there was a strong effect of IPI only at MCL therefore suggests that facilitation-like mechanisms are dominating at MCL but not at threshold, at least with our paradigm. This could also be due to the difference between the tasks rather than differences in neural mechanisms per se: loudness judgements at MCL rely on the response of a high number of neurons, while at threshold the results rely on detecting the spiking of a few neurons.

### Underlying Mechanisms at MCL

Results with paired pulses at MCL suggest a main contribution from the short-high phases (Figs. 4 and 5). For both polarities, paired pulses required less current than a single pulse in order to elicit the same loudness. The difference was largest at 0-μs IPI (equivalent to doubling the phase duration) and decreased with increasing gap. At the longest IPI tested here (172 μs), the MCLs of the paired pulses were smaller than that of the single pulses, and this difference was greater for cathodic than anodic stimulation (2.2 dB vs 0.9 dB, respectively).

**Fig. 7:**
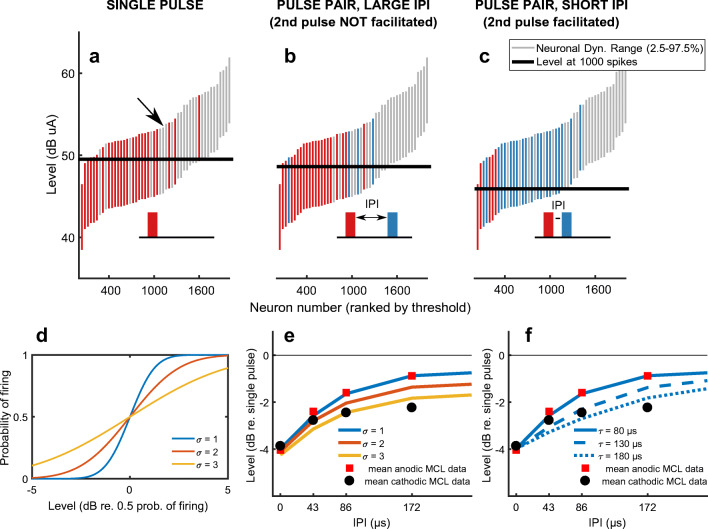
Modelling results. **A**–**C** show the dynamic range of 50 out of the 2000 neurons before stimulation (grey bars), after being recruited by the first/single pulse (red bars) and after being recruited by a combination of the first and second pulse (blue bars). The horizontal black lines in panels A to C indicate the level at which 1000 neurons are recruited. See text for more details. The insets are schematics of the short-high phases, corresponding to each condition. **D** shows the function linking stimulus level to probability of firing, based on a cumulative normal distribution with varied sigmas. A sigma of 2 was used for panels A to C. **E** shows the effect of changing sigma on the level yielding 1000 spikes at different IPIs. Mean results from our listeners are overlaid with squares and circles. **F** shows the effect of changing the time constant of the facilitation component in the model, with sigma fixed at 1

Figure 7 shows a conceptual model that may account for both the effect of IPI at MCL and the non-zero value at the longest IPIs. We first generate a population of 2000 neurons with thresholds following a Gaussian distribution on a logarithmic scale (to approximate data of cat recordings from Miller et al. 1999b). A subset of 50 of these neurons is shown in panels A to C of Fig. 7. The grey and coloured bars represent each neuron’s dynamic range, going from a probability of firing of 2.5 to 97.5 %. The size of these bars is identical across neurons, because we gave all neurons the same underlying function linking the probability of firing to the input level. For panels A to C, this function is a normal cumulative distribution with a standard deviation, σ, of 2 dB (panel D, in red).

We then find the level that results in 1000 spikes, which we will assume is equivalent to a given loudness. The red bars in panel A shows which neurons are recruited with a single pulse at the level indicated by the black horizontal thick line. One can notice that a certain amount of neurons whose dynamic range include this level have not spiked (e.g. neuron shown with black arrow). Thus, even without any facilitation, presenting a second pulse at the same level will give a second chance for these neurons to spike. The model presented here assumes that only neurons that did not fire to the first pulse can do so, because the longest inter-pulse interval tested here is much shorter than the absolute refractory period observed in physiological recordings and implemented in phenomenological models ((McKay and McDermott 1998; Miller et al. 2004; Boulet et al. 2016). This “second chance” effect is shown in panel B, which simulates a pulse pair with a long IPI (> 500 μs) where no facilitation occurs between the two pulses. The horizontal black line now lies at a lower level, at which about 800 neurons respond to the first pulse (red), with a further 200 responding to the second pulse (blue). This explains qualitatively the non-zero value we observed at the largest IPIs in our data at MCL, and has been suggested previously by McKay and McDermott (1998) in their loudness model.

To simulate facilitation at short IPIs, we reduce the thresholds of the neurons in response to the second pulse by an exponential factor as shown in Eq. 1.

1 $$ {THR}_{facilitated}=\frac{THR}{1+A.{e}^{\frac{- IPI}{\tau }}} $$

In Eq. 1, THR is the original threshold of the neurons, IPI the inter-pulse interval, *A* is the gain, and τ is the time constant. We set the gain to 0.6 to match the 4-dB reduction at 0-μs IPI observed in Fig. 4. The results are shown in panel C of Fig. 7. At the level denoted by the black horizontal line, the first pulse recruits only about 100 neurons. At the same level, due to facilitation, the second pulse recruits a further 900 neurons, leading to a total of 1000 neurons firing. The solid red line in panel E shows the level needed to elicit 1000 spikes, as a function of the IPI, using the model parameters implemented in panels A-C, namely, σ = 2 dB and τ = 80 μs. The different coloured lines of panel E shows the effect of changing σ, whereas the dashed and dotted blue lines of panel F show the effects of fixing σ at 1 dB and changing τ. This simple conceptual model can account for both the increase in MCL with increasing IPI and the non-zero value at the longest IPIs. The MCL data presented in Fig. 4 for the two different polarities are superimposed on the model predictions in panels E and F of Fig. 7 and will be discussed in the next section.

### Polarity Effects at MCL with the Pulse Pairs

As mentioned in the introduction, it has been suggested that changing the polarity from anodic to cathodic will target different portions of the SGNs (Miller et al. 1999a; Rattay et al. 2001), with cathodic stimulation targeting the more peripheral sites. Results from Cartee et al. (2006) in the cat also suggest longer time constants of facilitation for these peripheral sites. One could therefore interpret our larger cathodic reduction at the longest IPIs as a direct consequence of such longer time constant of integration from peripheral sites.

It is however not possible to conclude whether the differences we see between the anodic and cathodic results at MCL can be directly translated into differences in terms of time constant of charge integration at a neuronal level. In the model predictions plotted in Fig. 7, panels E and F, it can be seen that the anodic data (red squares) can be fit using σ = 1 dB and τ = 80 μs, but that there are at least two ways to fit the cathodic data (black circles). One of these (panel F) is to increase the time constant τ to 180 μs, which would be consistent with the portion of the SGN targeted by cathodic stimulation having a longer integrative time constant. This is in accordance with cathodic stimulation targeting more peripheral sites (Miller et al. 1999a; Rattay et al. 2001) and peripheral sites having longer time constants of facilitation (Cartee et al. 2006). However, as shown in panel E, a similar effect can be obtained without changing т but by assuming a shallower input-output function (σ = 3 dB). A shallower input-output function at a neuronal level also translates into a shallower input-output function at a population level with this simple model. A higher value of σ is therefore also consistent with the increased dynamic range we observed for the single cathodic pulses when compared with the anodic pulses (Fig. 2).

### Further Research Directions

Our paradigm seems more promising in measuring the charge integration properties of the SGNs at MCL than at detection threshold. This contrasts with most proposed psychophysical predictors of neural health that generally focus on the across-electrode variation in the effects of parameters such as inter-phase gap, pulse rate, and stimulus polarity, at detection threshold (Bierer and Faulkner 2010; Pfingst et al. 2015; Carlyon et al. 2018; Goehring et al. 2019; Jahn and Arenberg 2019a, b; Brochier et al. 2020; Mesnildrey et al. 2020). Although there might indeed be more across-electrode variability at threshold (because of stimulating more targeted populations of neurons), it is worth nothing that some of these measures correlate between threshold and MCL (e.g. polarity effects, Jahn and Arenberg 2019b) and could therefore still be informative at MCL. Furthermore, there are several benefits of having a measure at MCL, such as using levels that are more clinically relevant and the possibility of measuring physiological responses (eCAP/eABR.eASSR) to the single and paired pulses. The latter might not only be of interest for patients who cannot perform psychophysical tasks, but would also allow for comparison with animal models of polarity effects (e.g. cat, Miller et al. 1999a; mouse, Navntoft et al. 2020), where different pathologies can be induced artificially.

Finally, since the effect of IPI on MCL is monotonic, one could obtain a simple measure by comparing the MCLs (and/or eCAPs) obtained at IPIs of 0 and 172 μs, perhaps doing so both for anodic and cathodic pulses. This could then provide an efficient estimate of the characteristics of the neural membranes excited by a CI that could be used across electrodes, thereby aiding the interpretation of clinical results and their comparison with computer models and animal data. This is similar somewhat in concept to measuring the effect of increasing the inter-phase gap effect in biphasic pulses (Prado-Guitierrez et al. 2006; Ramekers et al. 2014) but importantly without any opposite-polarity interactions and allowing the effect to be measured separately for anodic and cathodic phases.

## Conclusion

We describe a paradigm consisting of pairs of pseudo-monophasic pulses where two short-high pulses with the same polarity followed each other. The aim was to study the temporal integration of currents in a polarity-specific manners while limiting the contribution from opposite-polarity currents. For both anodic and cathodic currents, changing the inter-pulse interval had a strong effect on the loudness, consistent with the hypothesis that the short-high phases dominated the neural response. Furthermore, this effect interacted with the polarity of the short-high phases. A simple conceptual model suggests that this might reflect differences in charge integration properties of the underlying SGN population. Results at threshold showed no effect of inter-pulse interval, nor an interaction with polarity, which might partly stem from the contribution of the long-low phases.
